# A synbiotic mixture of *Bifidobacterium breve* M16-V, oligosaccharides and pectin, enhances Short Chain Fatty Acid production and improves lung health in a preclinical model for pulmonary neutrophilia

**DOI:** 10.3389/fnut.2024.1371064

**Published:** 2024-06-28

**Authors:** Gillina F. G. Bezemer, Mara A. P. Diks, Esmaeil Mortaz, Ingrid van Ark, Jeroen van Bergenhenegouwen, Aletta D. Kraneveld, Gert Folkerts, Johan Garssen

**Affiliations:** ^1^Division of Pharmacology, Department of Pharmaceutical Sciences, Faculty of Science, Utrecht University, Utrecht, Netherlands; ^2^Impact Station, Hilversum, Netherlands; ^3^Department of Microbiology & Immunology, Lineberger Comprehensive Cancer Center, University of North Carolina at Chapel Hill, Chapel Hill, NC, United States; ^4^Respiratory Immunology Research Center, NRITLD, Shahid Beheshti University of Medical Sciences, Tehran, Iran; ^5^Danone, Nutricia Research BV, Immunology, Utrecht, Netherlands

**Keywords:** pulmonary neutrophilia, gut-lung axis, nutraceuticals, synbiotics, neutrophil to lymphocyte ratio, Short Chain Fatty Acids, acetate, butyrate

## Abstract

**Introduction:**

Pulmonary neutrophilia is a hallmark of numerous airway diseases including Chronic Obstructive Pulmonary Disease (COPD), Neutrophilic asthma, Acute Lung Injury (ALI), Acute Respiratory Distress Syndrome (ARDS) and COVID-19. The aim of the current study was to investigate the effect of dietary interventions on lung health in context of pulmonary neutrophilia.

**Methods:**

Male BALB/cByJ mice received 7 intra-nasal doses of either a vehicle or lipopolysaccharides (LPS). To study the effect of nutritional interventions they received 16 intra-gastric doses of either a vehicle (PBS) or the following supplements (1) probiotic *Bifidobacterium breve* (*B. breve*) M16-V; (2) a prebiotic fiber mixture of short-chain galacto-oligosaccharides, long-chain fructo-oligosaccharides, and low-viscosity pectin in a 9:1:2 ratio (scGOS/lcFOS/lvPectin); and (3) A synbiotic combination *B. breve* M16-V and scGOS/lcFOS/lvPectin. Parameters for lung health included lung function, lung morphology and lung inflammation. Parameters for systemic immunomodulation included levels of fecal short chain fatty acids and regulatory T cells.

**Results:**

The synbiotic supplement protected against the LPS induced decline in lung function (35% improved lung resistance at baseline *p* = 0.0002 and 25% at peak challenge, *p* = 0.0002), provided a significant relief from pulmonary neutrophilia (40.7% less neutrophils, *p* < 0.01) and improved the pulmonary neutrophil-to-lymphocyte ratio (NLR) by 55.3% (*p* = 0.0033). Supplements did not impact lung morphology in this specific experiment. LPS applied to the upper airways induced less fecal SCFAs production compared to mice that received PBS. The production of acetic acid between day −5 and day 16 was increased in all unchallenged mice (PBS-PBS *p* = 0.0003; PBS-Pro *p* < 0.0001; PBS-Pre, *p* = 0.0045; PBS-Syn, *p* = 0.0005) which upon LPS challenge was only observed in mice that received the synbiotic mixture of *B. breve* M16-V and GOS:FOS:lvPectin (*p* = 0.0003). A moderate correlation was found for butyric acid and lung function parameters and a weak correlation was found between acetic acid, butyric acid and propionic acid concentrations and NLR.

**Conclusion:**

This study suggests bidirectional gut lung cross-talk in a mouse model for pulmonary neutrophilia. Neutrophilic lung inflammation coexisted with attenuated levels of fecal SCFA. The beneficial effects of the synbiotic mixture of *B. breve* M16-V and GOS:FOS:lvPectin on lung health associated with enhanced levels of SCFAs.

## Graphical abstract

This study highlights bidirectional gut lung cross-talk in a mouse model for pulmonary neutrophilia. Repeated pulmonary challenge with LPS induced neutrophilic lung inflammation which was associated with a reduction in levels of fecal SCFA. Intra-gastric supplementation with the synbiotic mixture of *B. breve* M16-V and GOS:FOS:lvPectin induced enhanced levels of SCFAs and a positive impact on lung health indicated by improved lung function and improved neutrophil to lymphocyte ratio (NLR).
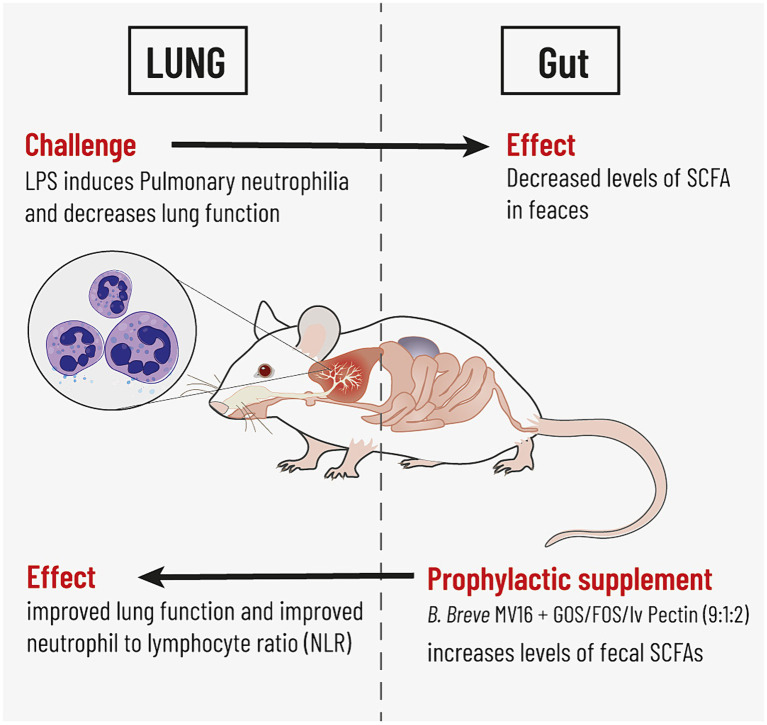

## 1 Introduction

Neutrophils are innate immune cells that play a key role in the first line of defense among others against invading pathogens. In a balanced situation, neutrophils undergo programmed cell death after their defensive actions, which when dysregulated, contributes to neutrophil accumulation, mucus hypersecretion, tissue destruction, airway remodeling and poor prognosis of certain lung diseases (1–4). Pulmonary neutrophilia is a hallmark of numerous airway diseases including, chronic obstructive pulmonary disease (COPD), pulmonary fibrosis, Acute Lung Injury (ALI), Adult Respiratory Distress Syndrome (ARDS) and certain types of severe persistent asthma (5–14). Such chronic respiratory diseases remain the leading causes of prevalence, mortality and disability-adjusted life years (DALY) with a high socioeconomic burden (15–17). COVID-19 is another recent example of an infection induced illness in which increased neutrophil to lymphocyte ratio (NLR) correlates with disease severity and mortality (18–22). With rising prevalence of chronic respiratory diseases and uncontrolled respiratory infections posing high socioeconomic burden, cost-effective solutions for maintaining respiratory health and reducing neutrophilic hyperinflammation are of utmost importance.

Throughout the last decades, evidence is accumulating that diet and gut health play important roles in maintaining effective and balanced immune responses and homeostasis in and beyond the gastro-intestinal tract (23, 24). The gut resides relatively high numbers of bacteria, from which a total microbial to human cell ratio of 1.3:1 can be estimated (25). The intestinal microbiota is crucial for human health and require human diet as chief source of energy for their growth (26). Diet has a large and temporal effect on gut microbiota composition which has implications for inflammation and autoimmune diseases. Pro-, pre-, and synbiotics, are considered appealing cost-effective gut microbiota modulators that could aid the management of allergic and infectious airway diseases, via the so-called “gut-lung axis” (27–30). Probiotics are defined as “live microorganisms which, when administered in adequate amounts, confer health benefits on the host.” (31). Dietary prebiotics are defined as “selectively fermented ingredients that result in specific changes in the composition and/or activity of the gastrointestinal microbiota, thus conferring benefits upon host cells” (26). The definition of a synbiotic was updated May 2019 to “a mixture comprising live microorganisms ánd substrate(s) selectively utilized by host microorganisms that confers a health benefit on the host,” in other words a combination of pro and prebiotics (32). Although the interaction between diet and immunology seems promising it is also complex and requires more information before disease-specific recommendations can be made (33).

Current scientific literature illustrates that modulation of the microbial gut ecosystem with pro-, pre- and synbiotics has a multi-facetted impact on pulmonary immune responses. Dampening of the immune system is broadly proven in the context of allergic eosinophilic asthma and boosting of the immune system is shown to enhance defense against respiratory infections (34). These apparently contradictory anti- and pro-inflammatory effects of gut microbiota changes can in part be explained Toll Like Receptor (TLR) activation (35). TLRs regulate the balance between different immune responses by recognizing specific pathogen associated molecular patterns (PAMPS) and other microbial-, or commensal associated molecular patterns (MAMPS or CAMPS) (35–40). Commensal gut bacteria boost gut-lung mediated innate immune activation in part via the activation of TLR4, which is demonstrated in defense against *E. coli* pneumonia (41). Although the benefit of TLR4 activation during certain infections is evident, the overzealous pulmonary activation of the TLR4 pathway is linked to excessive neutrophilic inflammation, locally and systemically (35, 42, 43). In the current study a TLR4 activating compound, Lipopolysaccharides (LPS), was used as a model agent to induce such hyperactivation of innate immunity leading to pulmonary neutrophilia (44). This model simulates TLR4 mediated neutrophilic lung inflammation which is illustrative of pathogens and cigarette smoke (45–53), ozone, nitrogen dioxide and traffic related air pollutants (54–57) and TLR4 mediated consequences in severe neutrophilic COVID-19 (58–64).

Relatively little is known about the immune modulating capacity of the gut lung axis in the context of a disease state that is characterized by such overzealous activation of innate immune pathways. Literature covering the effect of gut microbiota modulation on neutrophilic lung disorders is relatively scarce compared to the evidence supporting anti-allergic and pro-defensive (anti-infective) effects (65). It has previously been reported that supplementation with different Lactobacilli strains can increase an immune response in neutrophils indicated by enhanced neutrophil respiratory burst enzymes and nitric oxide production over a period of 60 days, which was stated to be strain dependent and to reach a maximum capacity within a window of stable health (66). In chronic diseases however, the interplay between microbiota and neutrophils is highly contextual and has been mentioned to have both disease worsening as well as improving capacity (67). A more recent review elaborated on the strain specificity of probiotics toward neutrophil recruitment, indicating an advanced need for thorough examination of probiotic effects on neutrophils in specific disease contexts (68). A careful consideration of the right ingredient for the right disease context asks for a more thorough investigation of gut modulating ingredients in context of pulmonary neutrophilia.

In the current study *Bifidobacterium breve* (*B. breve*) M16-V was selected which is a strain that originates from the gut of an infant and has emerged as a commercial probiotic supplement to help establish a favorable microbial gut ecosystem of infants (69). Colonization of mucosal surfaces by microbiota in early life occurs in parallel to the development and education of the mucosal immune system which has long-standing consequences on inflammatory diseases (70). Since the effects of microbiome modulation stretch into adulthood it reasons further investigations for more broad applications of dietary concepts originating from studies for infant health (71). *B. breve* strains can indeed help to fight acute respiratory infections and shorten the illness period (72). Existing data is however also supportive of the hypothesis that *B. breve* could have added value in the specific context of TLR4 mediated hyperinflammation. *B. breve* compared to other broadly used Lactobacilli strains, has been shown to exert immune inhibitory effects by activating TLR9, and TLR2 but not TLR4 explaining its relatively low pro-inflammatory profile (73, 74). It has also been shown that Bifidobacterium, including *B. breve* M16-V, has the ability to reduce the incidence of necrotizing enterocolitis (NEC) in part by down-regulating TLR4 signaling (69, 75).

This supports further investigation of the capacity of *B. breve* to dampen excessive LPS mediated innate immune activation. A previous study already revealed that *B. breve* can prevent the development of alveolar damage and right ventricle heart hypertrophy in an LPS induced mouse model for COPD (76). However, in that specific COPD model, *B. breve* did not have an effect on the numbers of neutrophils in broncho alveolar lavage fluid (BALF) in contrast to prophylactic treatment with a prebiotic scGOS/lcFOS formula, that was shown to reduce alveolar damage, heart hypertrophy as well as neutrophilic inflammation (76).

A beneficial effect of *B. breve* M16-V along the gut-lung axis has also been shown in murine allergic models illustrating its ability to dampen chronic eosinophilic airway inflammation to the same extend as the widely used inhalation corticosteroid, budesonide (73). *B. breve* M-16 V combined with short-chain galacto-oligosaccharides and long-chain fructo-oligosaccharides (scGOS/lcFOS) resulted in a preventive effect on asthma-like symptoms in infants and possibly on subsequent development of asthma (77, 78). This scGos/lcFos formula was originally developed to mimic the function and structure of oligosaccharides from breast milk and has proven benefits on baby’s health including the reduced occurrence of infections and reduced incidence of allergic symptoms (77, 79).

In the current study design a prebiotic fiber mixture was included consisting of scGOS/lvFOS further supplemented with lvPectin (mimicking the acidic fraction of human milk oligosachcharides) which are plant cell-wall polysaccharides that can be metabolized by commensal bacteria in the gut, including *Bifidobacterium* (80). To test the value of such a mixture in context of pulmonary neutrophilia, a synbiotic formula of *B. breve* M16-V combined with scGOS/lcFOS/lvPectin was included in the study design as well. Furthermore, a functional parameter for lung health was added next to immunological and morphological lung parameters. Finally, the current study elaborated on associations between gut and lung by analyzing the effect of inhaled LPS and of the dietary supplements on Short Chain Fatty Acid (SCFA) production. SCFA are gut microbe-derived metabolites that act as key mediators affecting the direction of the local and systemic immune system (30). Breastmilk and/or formula with probiotics/prebiotics could modulate toward more favorable microbial species producing different amounts of SCFAs exerting anti-inflammatory effects which has been linked to improve microbial dysregulation associated with increased TLR4 signaling in preterm infants (81). SCFAs have previously also been linked to a protective effect of enteral diets in context of elastase-induced lung inflammation and emphysema (82).

## 2 Materials and methods

### 2.1 Animals

136 Male BALB/cByJ mice, 6–8 weeks of age were obtained from Charles River Laboratories. Mice were group housed under controlled conditions (temp 20°C, humidity 40–60%, and an inversed 12-h light–dark cycle). Cage bedding was enriched with tissue and non-toxic pvc pipes. Mice were given *ad libitum* access to standard food (801730 CRM (E) Expanded Special Diets Services, England) and water. Mice were randomly divided over the different study groups and were allowed to adapt for 2 weeks before the start of the experiments. Clinical appearance was checked throughout the study by measuring body weight at least 3 times per week and by scoring vital signs daily. Scoring categories included: normal indicated as “0”; moderate discomfort indicated as “1”; and severe discomfort indicated as “2.” Scoring indicators included any changes in behavior (abnormal breathing/chest tightness, excessive salivation, immobility, shaking and tremors, continuous convulsion, inability to respond to stimuli, changes in social grooming, self-mutilation) and external appearance (pilo erection fur, abnormal posture, injuries). All animal studies were approved by the Utrecht Universities Committee on Animal Research (DECnumber: 2011.II.02.044) and comply with the principles of 3R. The animal experiments are carried out in accordance with (inter) national guidelines of animal experiments.

### 2.2 Experimental design LPS model and intra-gastric supplements

**Figure 1:**
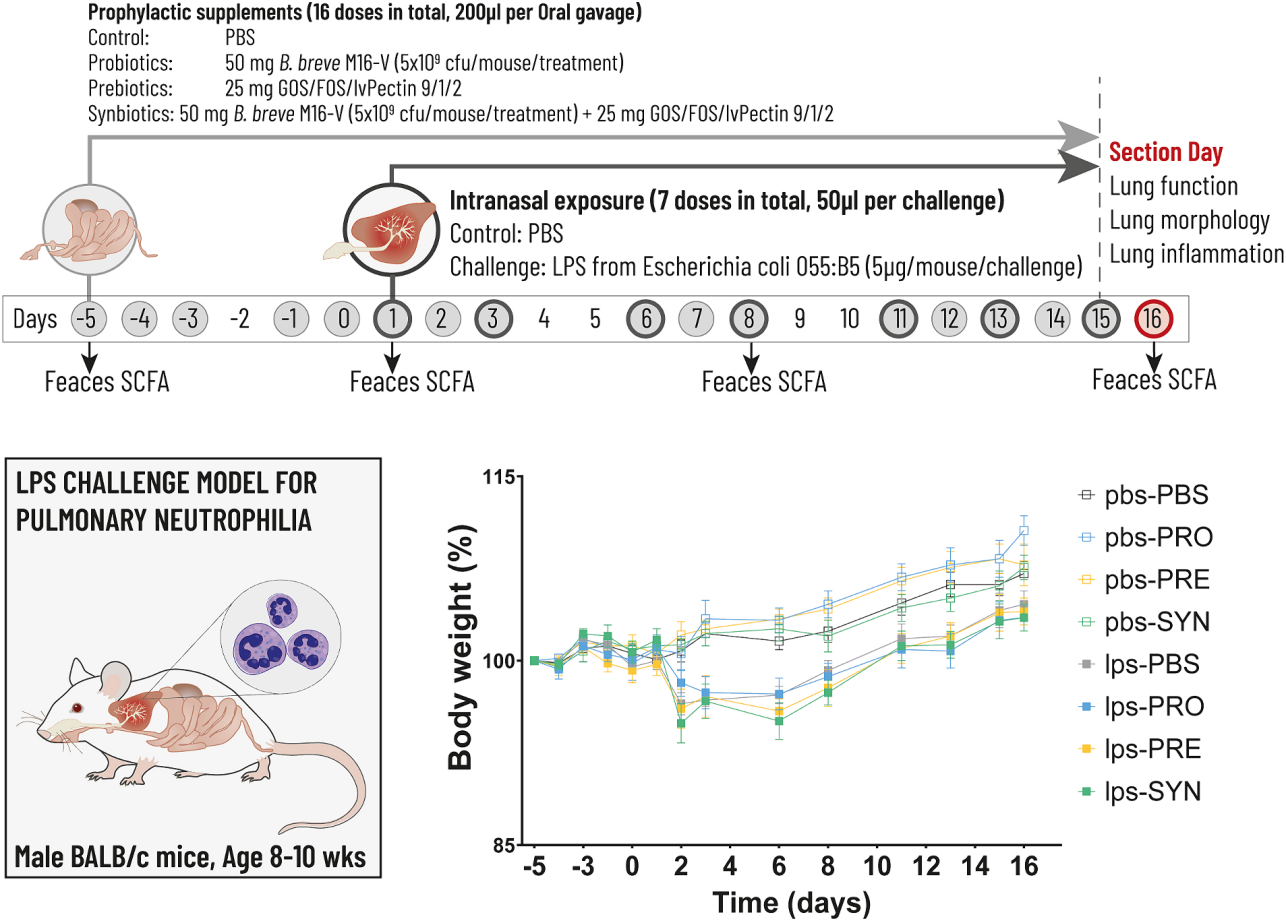
The timeline (above) depicts the experimental design for the LPS induced mouse model for pulmonary neutrophilia. The timeline shows the days on which the airways were challenged with either LPS or PBS control (dark grey circles) and the days on which the intragastric prophylactic supplements were provided (solid light grey circles). The arrows indicate the 4 timepoints at which feces was collected for the analysis of Short Chain Fatty Acids (SCFA). Lung function, lung morphology and lung inflammation was analyzed at day 16 (red circle). The graph (below) depicts body weight which was analyzed throughout the experiment as key indicator for mouse well-being. Stunted growth as a result of the first LPS challenge remained within acceptable levels of discomfort reaching a max of 3.5%, the day after the first LPS challenge (n.s.).

An overview of the experiment is presented in Figure 1. Male BALB/cByJ mice were instilled intra nasally (i.n.) under isoflurane anesthesia with 50 μL phosphate-buffered saline (PBS) or LPS (5 μg/mouse/challenge, dissolved in 50 ul PBS) purified from *Escherichia coli* O55:B5 (Sigma-Aldrich, Zwijndrecht, the Netherlands) which was administered 7 times in total, spread over a period of 15 days. Starting 1 week prior to the first LPS exposure until the end of the experiment, mice received 5 oral gavages per week to provide intra-gastric supplementations. Control groups (12 to 20 mice per group) received 200 ul of a vehicle (PBS) and study groups (20 mice per group) received 200ul of a solution (pH7) comprising one of the 3 dietary formula: (1) 25 mg of a prebiotic fiber mixture of GOS:FOS:lvPectin in a 9:1:2 ratio; (2) 50 mg of the probiotic *B. breve* M16-V (5×10^9 cfu/dose); and (3) A synbiotic combination of 25 mg GOS:FOS:lvPectin 9:1:2 and 50 mg *B. breve* M16-V (5×10^9 cfu/dose). Formulations were prepared fresh daily, short before enteric administration because of the oxygen sensitivity of *Bifidobacteria*. At the end of the experiment, at day 16, mice were euthanized, with an overdose of 600 mg/kg body weight sodium pentobarbital, i.p. (Nembutal™, Ceva Santé Animale BV, Naaldwijk, the Netherlands) and exsanguinated by cardiac puncture after which biological samples were obtained for further analysis.

### 2.3 Measurement of basal lung function *in vivo*

Per study group, 12 mice were reserved for the analysis of airway function, which was measured using an invasive EMKA plethysmography system (EMKA technologies) as described previously (83). Briefly, 24 h after the final exposure, mice were anesthetized via i.p. injection with a mix containing Ketamine (Vetoquinol S.A., France; 125 mg/kg) and Medetomidine (Pfizer, Netherlands; 0.4 mg/kg). Body temperature was kept at 37°C by placing the mice on a heating pad. Mice received a trachea cannula for mechanical ventilation with O2/air (1, 2). Mimicking spontaneous breathing, ventilation frequency was set at 150 breaths/min and tidal volume at 0.3 mL. Mice were placed in individual body boxes equipped with a pressure transducer (EMKA Technologies, Paris, France). For measuring transpulmonary pressure, the signal was obtained via a probe inserted alongside the trachea via the esophagus. Airflow and tidal volume (V_t_) were determined using a flow transducer able to measure flow fluctuations inside the body box. V_t_ is expressed in ml. The lung resistance (R_L_) was obtained by dividing the transpulmonary pressure by airflow at isovolume points (measured for 3 min) (cmH2O/ml/s). After measurement of basal lung function, 7 increasing doses of methacholine (acetyl-β-methyl-choline chloride, Sigma) (0–25 mg/mL, 10% puff for 10 s) were administered by aerosol generated in a nebulizer (EMKA Technologies, Paris, France) connected in between the animal in the body box and the ventilator (EMKA Technologies, Paris, France). After each dose of methacholine, V_t_ and R_L_ were measured for 3 min. After the final dose of Metacholine, mice were euthanized by an intraperitoneal overdose of sodium pentobarbital (Nembutal™, Ceva Santé Animale, Naaldwijk, The Netherlands, 600 mg/kg BW) followed by exsanguination by heartpuncture.

### 2.4 Measurement of cellular and cytokine parameters in BALF

Directly after airway function analysis and exsanguination, the lungs of 12 mice per experimental group were lavaged 4 times *in situ* with each 1 mL of pyrogen-free 0.9% w/v saline (37°C) via a cannula that was inserted into the trachea. The first ml of lavage fluid was supplemented with a protease inhibitor cocktail (Complete mini, Roche applied sciences) to avoid degradation of protein components. All BALF samples were kept on ice until cells were pelleted at 400 g for 5 min at 4°C. Supernatants were discarded except from the first lavage, which was aliquoted and stored at -80°C until further analysis for keratinocyte-derived chemokine (KC), Granulocyte Macrophage-Colony Stimulating Factor (GM-CSF), Macrophage Inflammatory Protein-1α (MIP1a), mouse Interleukin-23 (p19/p40) (IL-23 P19/P40) using a Cytometric Bead Array (CBA) flex set according to standard instruction manual (BDTM CBA flex sets: instrument Setup, Data acquisition, and analysis) using BD FACSCanto. The BALF cell pellets were resuspended in 150 μL of cold 0.9% w/v normal saline and pooled per animal. Total cell numbers and viability were assessed by trypan blue exclusion using a Burker-Türk chamber. For differential cell counts, 5×10^4^ cells per BALF sample were cytospinned for 5 min at 400 g, then airdried and stained with a Diff-Quik staining set (Dade A.G., Düdingen, Switzerland). A total of 200 cells were counted from each slide for differential cellular categorization in macrophages, neutrophils and lymphocytes based on morphological differences.

### 2.5 Measurement of FoxP3+ regulatory T cells

An exploratory analysis of the presence of FoxP3+ regulatory T cells (FoxP3 Treg) in thoracic – and mesenteric lymph nodes and in spleen was performed by multi-parameter flow cytometry. Freshly isolated spleens and lymph nodes were weighed after which in 5 mice per group the cells were isolated using a cell strainer. Red blood cells were lysed and cells were washed after which they were suspended in standard Fluorescence Activated Cell Sorting (FACS) buffer (PBS/1% Bovine serum albumin (BSA)). Cells were incubated at a density of 1×105 cells/100 μL per well with the following anti-mouse antibodies from ebioscience: anti-mouse FoxP3-APC, CD4-FITC and CD25-PE. Isotype controls were used as indicated in the manufacturers protocol: Rat Immunoglobulin G (IgG)2b, k and IgG2a, k (ebioscience 17-4321-41), Armenian hamster IgG Isotype (ebioscience 11-4888-81 and 12-4888-81). Cells were washed to remove the unbound antibodies and resuspended in PBS/1%BSA prior to analysis on a FACS Canto II using CellQuest software (BD Biosciences, the Netherlands). For the analysis of forward angle light scatter, side angle light scatter, and cell surface receptor expression, data were acquired in real time as percent (%) positive-expressing cells and geometric mean fluorescence intensity (MFI).

### 2.6 Lung morphometric and histological analysis

Since both lung function analysis and BALF retrieval influence the airway architecture, separate mice, (8 per study group plus 1 PBS-PBS control group), were used for purposes of lung morphometric analysis. After exsanguination, a cannula was placed in the trachea and the lungs were taken out en bloc. The lungs were inflated with neutral 10% formalin under constant pressure (25 cm fixative) for five minutes. Tracheas were sutured and lungs were immersed in fresh fixative until completely degassed after which the lungs were changed to 70% ethanol. Left lungs were further dehydrated and embedded in paraffin blocks. Five μm sections were cut and samples at various tissue depths (200 μm, 400 μm, 600 μm and 800 μm) were mounted on coated slides after which the tissue was rehydrated and stained with Mayer hematoxylin/eosin (H&E). In order to analyze changes in tissue morphology, six photo-microscopic images were made per section at a total magnification of 10 times10x. Inter alveolar distance was measured by the mean linear intercept (Lm). Microscopic images were projected onto a reference grid using Image Pro MC Plus 7.0 software. Lm was expressed as the total grid length in μm divided by the number of alveolar wall-grid line intersections. A higher Lm-score reflects more disruption of alveolar walls which is an indication for emphysematous damage.

### 2.7 Short chain fatty acids (SCFAs)

At 4 time points throughout the experiment, 7 mice per experimental group were shortly separated for individual dry fecal pellet collection (Figure 1). Urine was absorbed using tissue as cage bedding. Two pellets per animal were dissolved immediately after collection at a dilution of 0.11 g feces/ml PBS and stored at −80 until quantitative SCFA analysis by a Varian 3,800 gas chromatograph (GC) (Varian, Inc., Walnut Creek, U.S.A.) equipped with a flame ionization detector as described before (84). Briefly, 350 μL of fecal suspension was mixed with 200 μL 5% (v/v) formic acid, 100 μL 1.25 g/L 2-ethylbutyric acid (as internal standard, Sigma-Aldrich, Zwijndrecht, The Netherlands) and 350 μL MilliQ water. The samples were centrifuged for 5 min at 16,000 × *g* to remove large particles. The supernatant was collected of which, 0.5 μL was injected at 80°C in the column (Stabilwax, 15 m × 0.53 mm, film thickness 1.00 μm, Restek Co., USA) using helium as carrier gas (3.0 psi). Next, the oven was heated at a speed of 16°C/min to 160°C, followed by heating at a speed of 20°C/min to 220°C at which the temperature was maintained for 1.5 min. The temperature of the injector and the detector was 200°C. Levels of acetic acid, propionic acid and butyric acid are expressed in μmol/g of fecal weight.

### 2.8 Statistical analysis

Data are expressed as mean ± standard error of mean (SEM). Comparisons between data were tested using GraphPad Prism version 9.1. Methods used included descriptive statistics; ordinary one way analysis of variance (1 way ANOVA) with Bonferroni’s multiple comparisons test; Tukey’s multiple comparison test, with individual variances computed for each comparison (2 way ANOVA); Simple lineair regression; and Unpaired t test; and Pearson correlation. Data were considered statistically significant with an *p* value smaller than 0.05. To label the strength of the Pearson associations, the r values between, 0–0.3 (or −0.3) are considered as weak, 0.3–0.7 (or −0.7) as moderate, 0.7–1.0 (or −1) as a strong positive (or negative) correlations (85).

## 3 Results

### 3.1 Animal weights and vital signs

Mouse vital signs were checked on a daily basis and weights were measured at least 3 times per week. During the adaptation phase, minor fight wounds (score of “1” in the well-being diary) occurred in 2 groups, which stabilized and did not reason for individual housing. During the gavage procedure damage was induced to the esophagus in 2 individual cases resulting in weight loss exceeding 15% of total body weight and a score of “2” in the well-being diary due to changes in social grooming and abnormal posture to avoid unnecessary further pain or distress these mice were euthanized immediately by cervical dislocation. Apart from these 2 cases, vital signs remained normal (score “0”) in all study groups throughout the experiment. The intranasal LPS challenge induced an attenuation of the normal body weight increase in all groups, which is a signal for the expected illness driving effect of LPS (Figure 1). The drop in body weight reached 3.5%, the day after the first LPS challenge, after which growth resumed. The impact of LPS on bodyweight was not significant and remained within reasonable levels of discomfort. The intra-gastric supplementations did not influence vital signs or bodyweight, which is a supportive indication for the tolerability of the oral supplements.

### 3.2 Airway function

**Figure 2:**
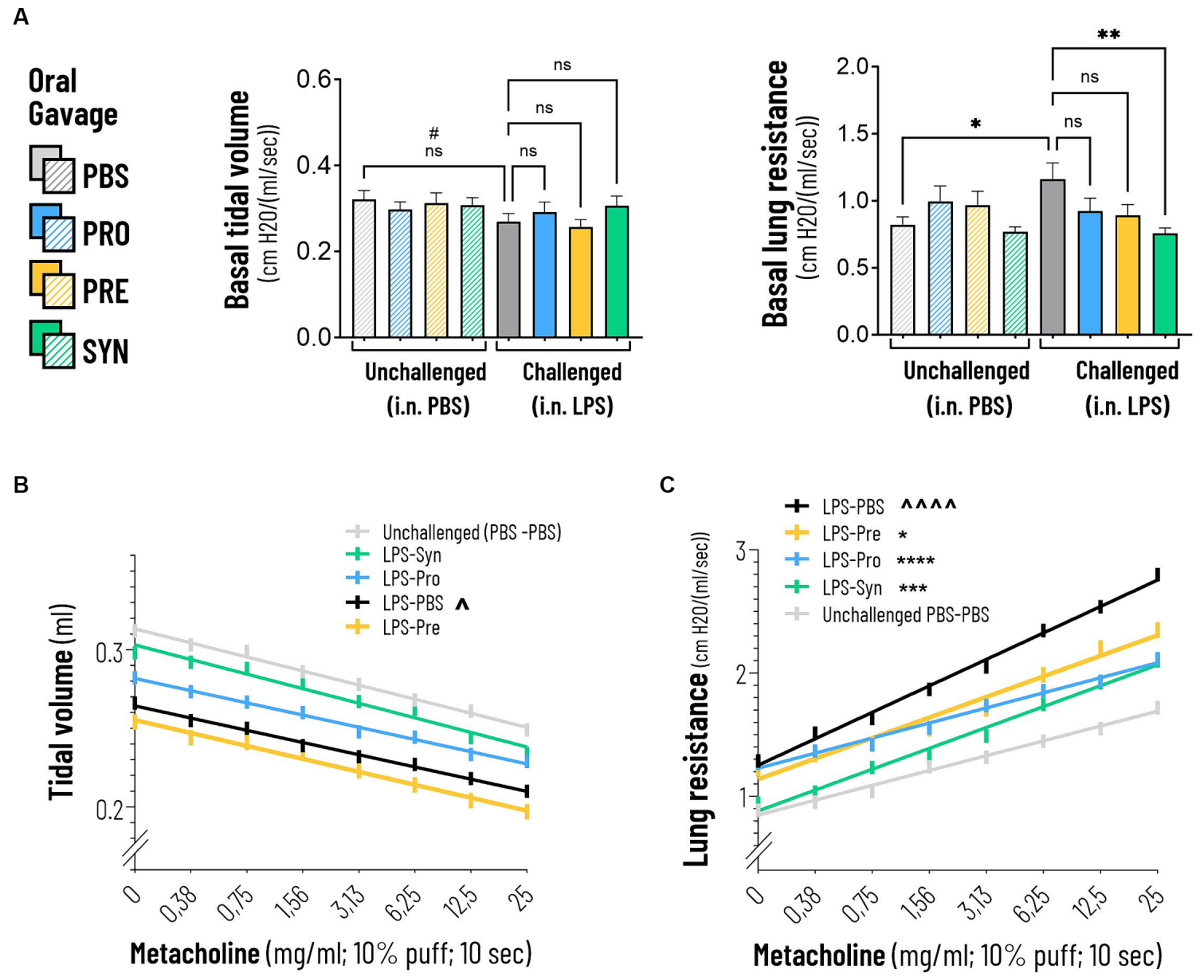
**(A)** Basal tidal volume was not impacted by intranasal LPS challenge. Lung resistance was negatively impacted by the intranasal LPS challenge at baseline and at increasing doses of Metacholine (indicated by ****p < 0.0001). The synbiotic mixture of *B. breve* M16-V and GOS:FOS:lvPectin significantly dampened the negative effect of LPS on lung resistance by 34.9% at baseline and 25% at peak challenge (indicated by ***p < 0.001 throughout the challenge). **(B)** The linear regression graph presents the pattern that step-wise methacholine challenge induces on the tidal Volume. A significant change (indicated by ^*p* = 0.0093) was seen between the effect of methacholine on tidal volume of the LPS-placebo and PBS-placebo. A significant treatment effect on tidal volume was not observed. **(C)** Simple linear regression analysis confirmed a strong significant change between the lung resistance dose–response curves of the PBS-placebo and LPS-placebo groups (indicated by ^^^^*p* < 0.0001). A treatment effect of synbiotics was also observed within the LPS challenged groups (indicated by ***p = 0.001). The lineair regression analysis furthermore pointed toward indications for a beneficial treatment effect of Probiotics, *p* < 0.0001 and Prebiotics *p* = 0.0042 on altering the effect of methacholine compared to mice that received a placebo supplement.

Lung function was analyzed in anesthetized and mechanically ventilated mice, by an EMKA plethysmography system. At baseline, LPS induced a modest decrease in tidal volume of 16.2% compared to mice that received i.n. PBS (*p* = 0.0019) (Table 1). The difference between LPS and PBS remained at a modest decrease of 15.7% (*p* = 0.0019) after exposure to the highest concentration of Metacholine. Tidal volume was not impacted by any of the treatments at basement level nor at increased levels of Methacholine. At baseline, LPS induced a robust effect on lung resistance (41.5% increase, *p* < 0.0001) compared to mice that received i.n. PBS (Table 2; Figure 2A). The synbiotic mixture of *B. breve* M16-V and GOS:FOS:lvPectin significantly dampened the negative effect of LPS on lung resistance by 34.9% at baseline (*p* = 0.0099). Metacholine dose response did trigger further worsening of the LPS induced adverse effect on lung resistance, reaching a 62% increase compared to the PBS control group (*p* < 0.0001, Figure 2A). The synbiotic mixture of *B. breve* M16-V and GOS:FOS:lvPectin was the only supplement that provided a significant decrease of the LPS induced deterioration in airway resistance up to the highest concentration of metacholine (*p* < 0.0002, Figure 2A). Simple linear regression analysis confirmed the change between the tidal volume metacholine dose–response curves LPS-placebo and PBS-placebo (*p* = 0.0093) and absence of a treatment effect within the LPS challenged groups (Figure 2B). Simple linear regression analysis also confirmed a strong significant change between the lung resistance dose–response curves of the PBS-placebo and LPS-placebo groups (*p* < 0.0001) as well as a treatment effect of synbiotics within the LPS challenged groups (*p* = 0.001) (Figure 2C). The linear regression analysis furthermore pointed toward indications for a treatment effect of probiotics, *p* < 0.0001 and prebiotics *p* = 0.0042 on altering the effect of the methacholine stressor compared to mice that received a placebo supplement.

**Table 1:** Overview of airway function, tidal volume data.

Tidal volume										
	PBS–PBS		LPS–PBS		LPS–PRO		LPS–PRE		LPS–SYN	
Basal function	Mean	SEM	Mean	SEM	Mean	SEM	Mean	SEM	Mean	SEM
	0.32	0.021	0.27	0.019	0.29	0.024	0.26	0.017	0.31	0.023
Challenge effect (Difference PBS-PBS)	-		−16.2%^**^ p = 0.0019		-					
Treatment effect (Difference LPS-PBS)	-		-		8.21^n.s.^		−4.46^n.s.^		13.76^n.s.^	
Metacholine (mg/mL; 10% puff; 10s)										
	Mean	SEM	Mean	SEM	Mean	SEM	Mean	SEM	Mean	SEM
0	0.31	0.020	0.27	0.018	0.28	0.022	0.25	0.016	0.30	0.022
0.38	0.30	0.021	0.26	0.016	0.27	0.022	0.24	0.016	0.29	0.022
0.75	0.30	0.021	0.25	0.018	0.27	0.020	0.24	0.016	0.29	0.022
1.56	0.29	0.021	0.24	0.017	0.26	0.020	0.24	0.016	0.28	0.022
3.13	0.28	0.022	0.23	0.019	0.25	0.019	0.22	0.015	0.27	0.024
6.25	0.27	0.022	0.23	0.019	0.24	0.019	0.21	0.015	0.26	0.024
12.5	0.26	0.023	0.22	0.019	0.23	0.018	0.20	0.015	0.24	0.025
25	0.25	0.022	0.21	0.019	0.23	0.017	0.20	0.017	0.23	0.025
Max Challenge effect (Difference PBS-PBS)	-		−15.66% n.s.		-					
Max Treatment effect (Difference LPS-PBS)	-		-		9.00^n.s.^		-6.19^n.s.^		11.43^n.s.^	
Simple linear regression metacholine dose response										
Equation	Y = −0.00895*X + 0.313		Y = −0.00777*X + 0.264		Y = −0.00777*X + 0.282		Y = −0.00824*X + 0.255		Y = −0.00927*X + 0.303	
Goodness of Fit (R squared)	99.35%		99.42%		99.23%		98.63%		96.74%	
Challenge effect (Difference PBS-PBS)	-		*p* = 0.0093		-					
Treatment effect (Difference LPS-PBS)	-		-		n.s.		n.s.		n.s.	

ns *p* > 0.05; *p ≤ 0.05; **p ≤ 0.01; ***p ≤ 0.001; ****p ≤ 0.0001.

**Table 2:** Overview of airway function, lung resistance data.

Lung resistance										
	PBS–PBS		LPS–PBS		LPS–PRO		LPS–PRE		LPS–SYN	
Basal function	Mean	SEM	Mean	SEM	Mean	SEM	Mean	SEM	Mean	SEM
	0.82	0.056	1.16	0.12	0.92	0.097	0.89	0.08	0.76	0.041
Challenge effect (Difference PBS-PBS)	-		41.47% **** p < 0.0001		-					
Treatment effect (Difference LPS-PBS)	-		-		−20.67 ^n.s.^		−23.17 ^n.s.^		−34.94*** *p* = 0.0002	
Metacholine (mg/ml; 10% puff; 10s)										
	Mean	SEM	Mean	SEM	Mean	SEM	Mean	SEM	Mean	SEM
0	0.89	0.06	1.29	0.13	1.27	0.22	1.23	0.21	0.94	0.12
0.38	0.95	0.07	1.51	0.17	1.37	0.25	1.34	0.22	1.03	0.16
0.75	1.04	0.07	1.63	0.18	1.42	0.20	1.42	0.25	1.23	0.21
1.56	1.24	0.10	1.87	0.20	1.55	0.21	1.55	0.26	1.32	0.21
3.13	1.32	0.11	2.05	0.21	1.74	0.27	1.71	0.26	1.49	0.21
6.25	1.45	0.13	2.34	0.23	1.86	0.26	1.98	0.29	1.75	0.20
12.5	1.55	0.14	2.54	0.24	1.93	0.25	2.20	0.30	1.92	0.21
25	1.72	0.14	2.80	0.31	2.12	0.30	2.35	0.31	2.10	0.23
Max Challenge effect (Difference PBS-PBS)	-		62.77%**** p < 0.0001		-					
Max Treatment effect (Difference LPS-PBS)	-		-		−24.41% ^n.s.^		−16.08% ^n.s.^		−24.98%*** p = 0.0002	
Simple linear regression										
Equation	Y = 0.1203*X + 0.8483		Y = 0.2146*X + 1.252		Y = 0.1223*X + 1.228		Y = 0.1668*X + 1.139		Y = 0.1693*X + 0.8811	
Goodness of Fit (R squared)	98.94%		99.36%		98.61%		97.18%		98.75%	
Challenge effect (Difference PBS-PBS)	-		*p* < 0.0001							
Treatment effect (Difference LPS-PBS)	-		-		*p* < 0.0001		*p* = 0.0042		*p* = 0.001	

ns *p* > 0.05; *p ≤ 0.05; **p ≤ 0.01; ***p ≤ 0.001; ****p ≤ 0.0001.

### 3.3 Lung morphology

**Figure 3:**
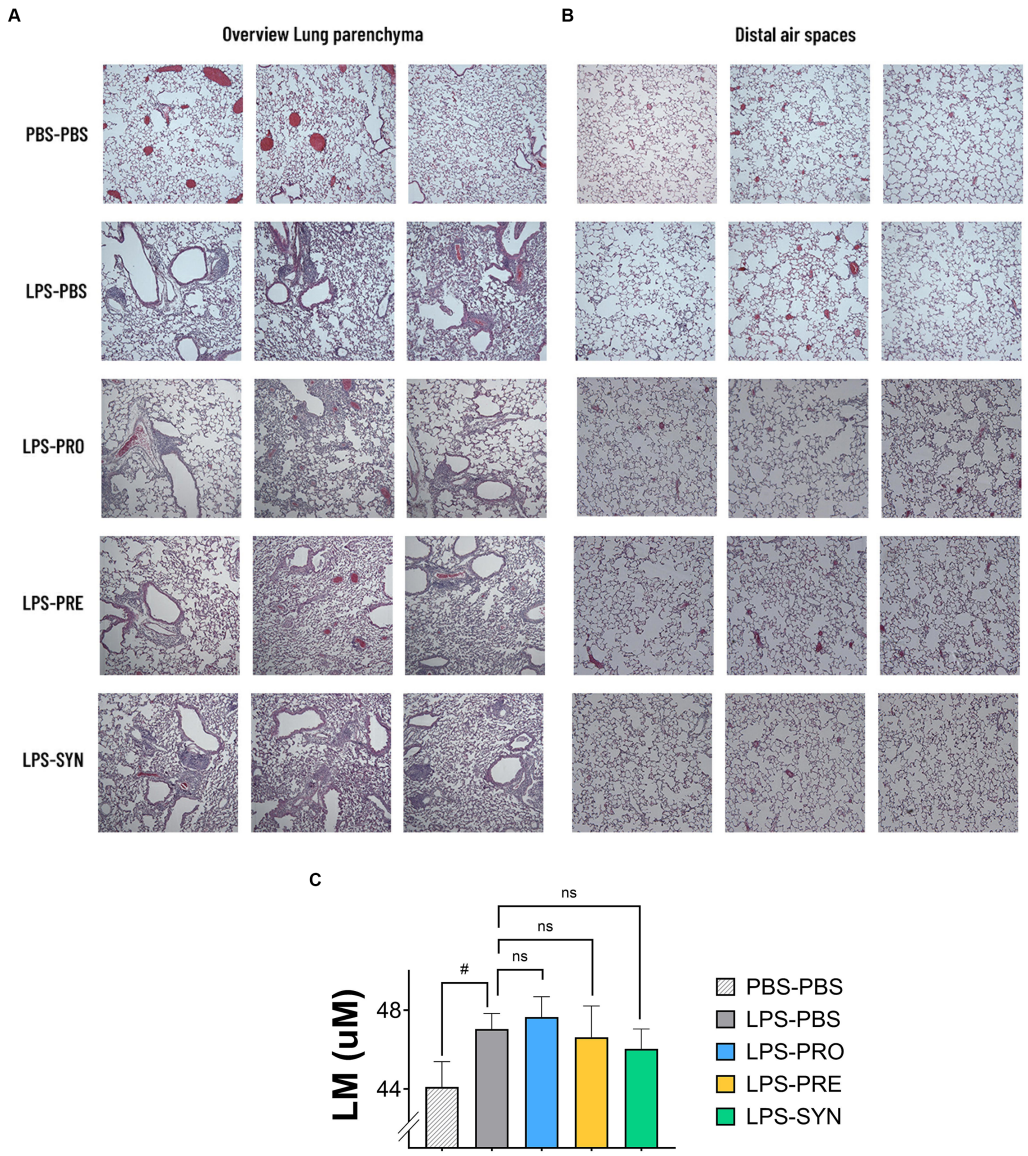
**(A)** Representative micrographs of H&E-stained sections of lung parenchyma of 3 different mice per group are shown. All LPS challenged groups showed areas of inflammation and remodeling of the lung parenchyma which was absent in mice that received i.n. PBS. The LPS effect includes cellular infiltrates intraluminal-, peribronchial-, and perivascular; thickening of bronchial-, and alveolar walls; and detachments of mucosal epithelium. **(B)** Representative micrographs of H&E-stained sections of the distal alveolar airspaces of 3 different mice per group are shown. The images depict larger open spaces in the LPS challenged groups which can be a signals for alveolar wall breakdown. **(C)** The graph depicts the calculated Lineair mean intercept (Lm score) as a measure of the disruption of alveolar walls. Lm was lowest in the PBS-PBS control group. The unpaired t test with Welch’s correction provided a *p*-value of 0.0469 for the difference between PBS-PBS and LPS-PBS. Of all tested treatments, the synbiotic group showed the lowest Lm-Score, which was however not significantly different from the LPS-PBS group.

Lung morphometric analysis was analyzed in distinct groups of mice, to avoid damage of the airway architecture as a result of lung ventilation or lavage procedures. H&E-stained lung tissue sections of all LPS challenged groups showed areas of inflammation and remodeling of the lung parenchyma which was absent in mice that received i.n. PBS. Representative micrographs of the lung parenchyma of 3 different mice per group are shown in Figure 3A. Images of the LPS challenged groups depict intraluminal-, peribronchial-, and perivascular cellular infiltrates, thickening of the bronchial-, and alveolar walls and detachments of mucosal epithelium. Figure 3B furthermore shows a selection of representative images of the distal alveolar airspaces of 3 different mice per group, depicting alveolar wall breakdown in the LPS challenged groups. The calculated Lm score, as indicator for emphysematous damage revealed little alterations between the different groups (Figure 3C). Lm was lowest in the PBS-PBS control group with a mean of 44.11 μm. The window of average Lm in the LPS groups ranged between 46.04 μm and 47.65 μm. An unpaired t test with Welch’s correction provided a modest difference between PBS-PBS and LPS-PBS (*p* = 0.0469). Of all tested treatments, the Synbiotic group showed the lowest Lm-Score (mean 46.04 μm), which was however not significantly different from the LPS-PBS group.

### 3.4 Pulmonary inflammation

**Figure 4:**
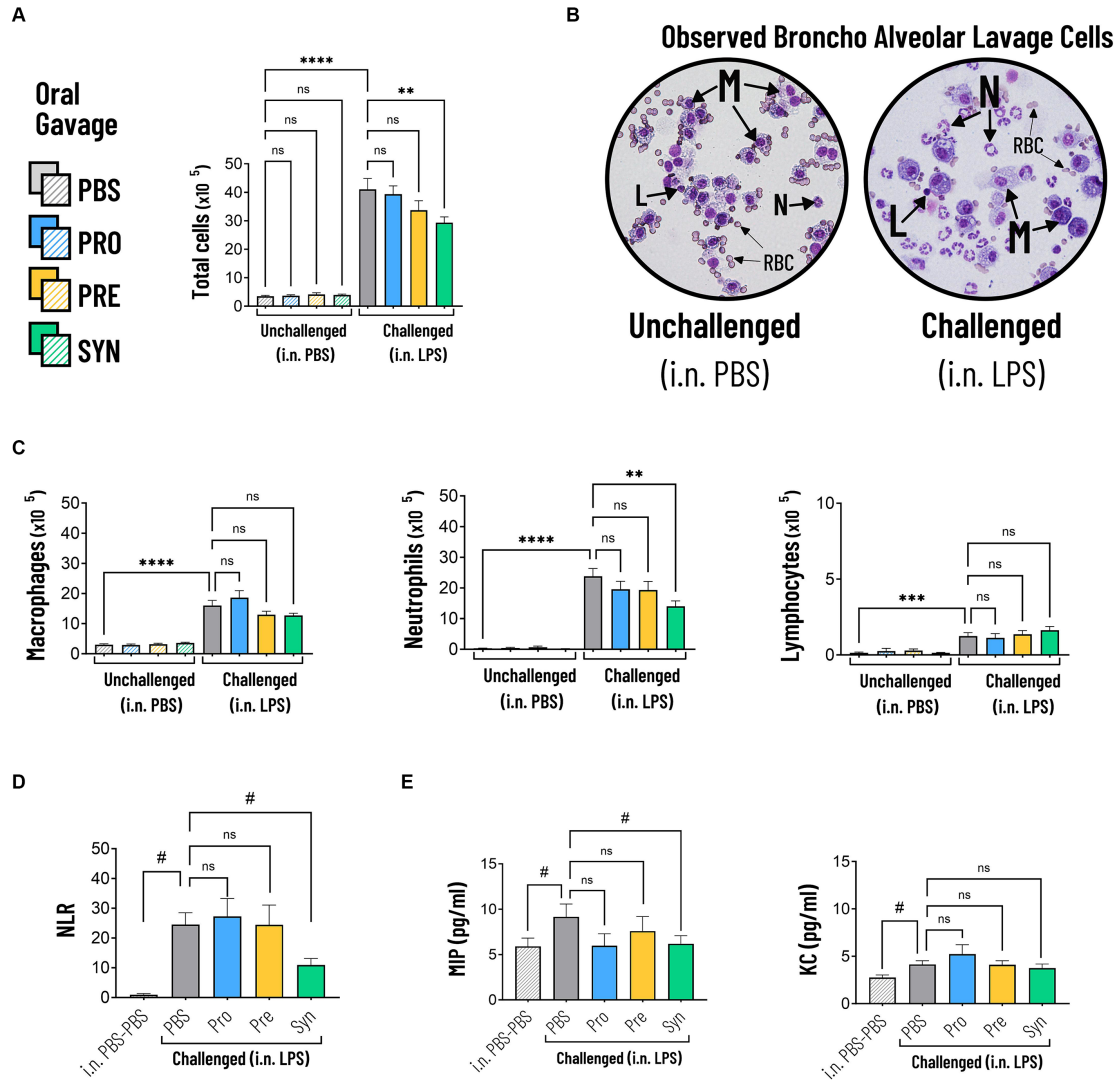
**(A)** The total amount of cells in BALF were significantly increased in mice that were challenged with LPS compared to PBS (indicated by ****p < 0.0001). The synbiotic mixture of *B. breve* M16-V and GOS:FOS:lvPectin significantly dampened the negative effect of LPS on total influx of cells by 28.6% compared to placebo (indicated by *p < 0.01). **(B)** Representative micrographs of Diff-Quik stained BAL cells showing the morphological differences between Macrophages (M), Lymphocytes (L), Neutrophils (N). Challenged mice showed high numbers of neutrophils which appear as cells with a typical multi-lobed nucleus and neutral-stained cytoplasmic granules. Red Blood Cells (RBC) were observed but not quantified. **(C)** LPS induced a significant increase in macrophages, neutrophils and lymphocytes compared to mice that received i.n. PBS (indicated by ****p < 0.0001). The synbiotic mixture of *B. breve* M16-V and GOS:FOS:lvPectin significantly reduced the numbers of neutrophils (indicated by **p < 0.01). **(D)** LPS induced a significant increase in the BALF Neutrophil to Lymphocyte Ratio (NLR) (indicated by #*p* < 0.0001, analyzed by *t* test). The synbiotic mixture of *B. breve* M16-V and GOS:FOS:lvPectin provided a significantly lower NLR compared to placebo (indicated by #*p* < 0.01). **(E)** BALF Cytokine analysis showed that LPS induced a 55.3% increase in MIP1a (indicated by #*p* = 0.0451) and a 49.6% increase in KC (indicated by #*p* = 0.0081). The synbiotic mixture of *B. breve* M16-V and GOS:FOS:lvPectin significantly dampened MIP1a (indicated by #*p* < 0.05) but not KC.

Intraluminal cellular and protein components were quantified in BALF (Figure 4). Challenging the airways with LPS induced significant increase in total numbers of cells, which was 10.7 fold higher compared to PBS control (*p* < 0.0001) (Table 3; Figure 4A). The synbiotic mixture of *B. breve* M16-V and GOS:FOS:lvPectin significantly dampened the negative effect of LPS on total influx of cells by 28.6% (*p* < 0.01). Differential analysis performed based on morphological differences of Diff-Quik stained BAL cells (Figure 4B) showed that LPS induced a significant upregulation of all quantified cells with a 4.3 fold increase in macrophages (*p* < 0.0001); 7.8 fold increase in lymphocytes (*p* < 0.0001); and 77.7 fold increase in neutrophils (*p* < 0.0001) compared to mice that received i.n. PBS (Figure 4C). In unchallenged mice, none of the analyzed enteric supplements influenced the differential amount of BAL cells. In the challenged mice however, the pro- pre- and synbiotic groups all presented lower neutrophil counts than the LPS-placebo group but a significant reduction in pulmonary neutrophilia was only observed in the synbiotic group. These mice that received *B. breve* M16-V and GOS:FOS:lvPectin had 40.7% less neutrophils (*p* < 0.01), 20.7% less macrophages (n.s.) and 30.6% more lymphocytes (n.s.) than the LPS-placebo group. These numbers are reflected in significantly improved pulmonary neutrophil-to-lymphocyte ratio of 55.3% (*p* = 0.0033) (Figure 4D). Figure 4E furthermore shows that the proinflammatory challenge effect of LPS was reflected by the analyzed cytokine profile showing a 55.3% increase in MIP (*p* = 0.0451) and a 49.6% increase in KC (*p* = 0.0081). The treatment effect was only in part reflected by the analyzed cytokine profile showing a significant decrease in MIP (32.3%, *p* = 0.0451) but only a slight non-significant decrease in KC of 9.3%. Table 3 provides a full overview of the lung inflammation data.

**Table 3:** Overview of lung inflammation data measured in Broncho Alveolar Lavage Fluid.

Lung Inflammation analyzed in Broncho Alveolar Lavage Fluid								
	PBS–PBS		PBS–PRO		PBS-PRE		PBS-SYN	
Cells	Mean	SEM	Mean	SEM	Mean	SEM	Mean	SEM
Total Cells	3.50	0.28	3.65	0.34	4.12	0.60	3.94	0.29
Treatment effect (Fractional change PBS-PBS)	-		0.04 ^n.s^		0.18 ^n.s^		0.13 ^n.s^	
Macrophages	3.04	0.25	2.90	0.35	3.13	0.34	3.57	0.28
Treatment effect (Fractional change PBS-PBS)	-		−0.05^n.s^		0.03 ^n.s^		0.17 ^n.s^	
Neutrophils	0.30	0.11	0.46	0.20	0.65	0.39	0.16	0.029
Treatment effect (Fractional change PBS-PBS)	-		0.5 ^n.s^		1.2 ^n.s^		−0.5 ^n.s^	
Lymphocytes	0.14	0.05	0.26	0.17	0.28	0.11	0.13	0.03
Treatment effect (Fractional change PBS-PBS)	-		0.8 ^n.s^		1.0 ^n.s^		−0.1 ^n.s^	
Cytokines	Mean	SEM						
MIP	5.89	0.914	n.a.					
KC	2.77	0.269	n.a.					
	LPS–PBS		LPS – PRO		LPS-PRE		LPS-SYN	
Cells	Mean	SEM	Mean	SEM	Mean	SEM	Mean	SEM
Total Cells	41.05	3.82	39.29	2.95	33.71	3.34	29.33	2.01
Challenge effect (fractional change PBS-PBS)	10.74****		-					
Treatment effect (% difference LPS-PBS)	-		−4.3 ^n.s^		−17.9 ^n.s^		−28.6**	
Macrophages	15.98	1.74	18.59	2.35	12.98	1.14	12.68	0.77
Challenge effect (Fractional change PBS-PBS)	4.25****		-					
Treatment effect (% difference LPS-PBS)	-		16.3 ^n.s^		−18.8 ^n.s^		−20.7 ^n.s^	
Neutrophils	23.61	2.73	19.57	2.58	19.32	2.81	14.01	1.78
Challenge effect (Fractional change PBS-PBS)	77.7****		-					
Treatment effect (% difference LPS-PBS)	-		−17.1 ^n.s^		−18.2 ^n.s^		−40.7**	
Lymphocytes	1.25	0.22	1.13	0.28	1.37	0.25	1.63	0.24
Challenge effect (Fractional change PBS-PBS)	7.8***		-					
Treatment effect (% difference LPS-PBS)	-		−9.4 ^n.s^		9.4 ^n.s^		30.6 ^n.s^	
NLR	24.52	3.95	27.23	6.01	24.39	6.67	10.94	2.22
Challenge effect (Fractional change PBS-PBS)	25.0****		-					
Treatment effect (% difference LPS-PBS)	-		11.0 ^n.s^		−0.5 ^n.s^		−55.3**	
Cytokines	Mean	SEM	Mean	SEM	Mean	SEM	Mean	SEM
MIP	9.15	1.42	5.98	1.32	7.59	1.60	6.19	0.89
Challenge effect (% change PBS-PBS)	55.3^#^		-					
Treatment effect (% difference LPS-PBS)	-		−34.7 ^n.s^		−17.1 ^n.s^		−32.3^#^	
KC	4.14	0.39	5.22	1.00	4.09	0.44	3.75	0.43
Challenge effect (% change PBS-PBS)	49.6^#^		-					
Treatment effect (% difference LPS-PBS)	-		26.1 ^n.s^		−1.2 ^n.s^		−9.3 ^n.s^	

ns *p* > 0.05; *p ≤ 0.05; **p ≤ 0.01; ***p ≤ 0.001; ****p ≤ 0.0001.

### 3.5 FoxP3+ regulatory T cells

**Figure 5:**
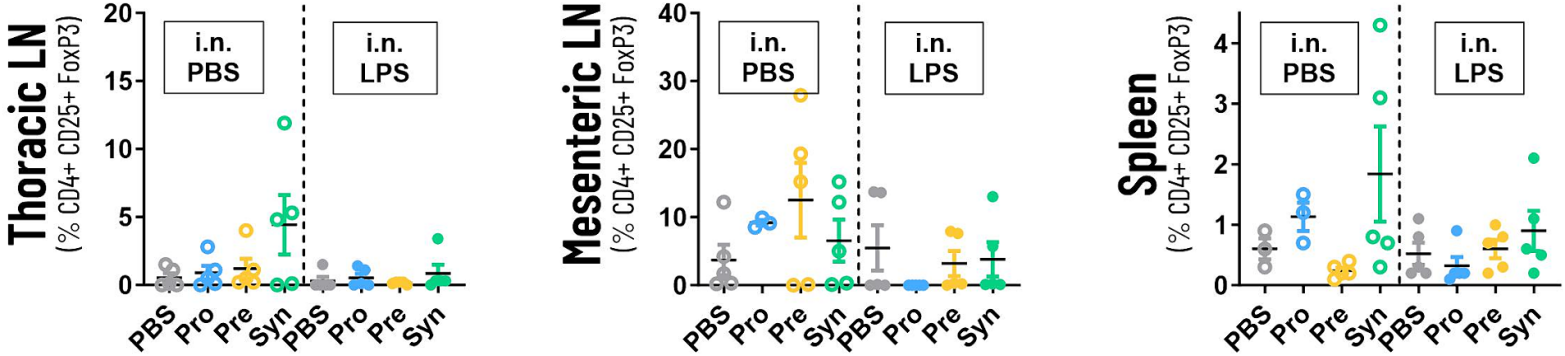
The graphs depicts the analysis of the numbers of systemic Tregs in thoracic lympnodes, mesenteric lympnodes and spleen tissue. The highest numbers of Tregs were counted in thoracic lympnodes and spleen of the mice that received the synbiotic formula of B *Breve* M16-V and GOS:FOS:lvPectin 9:1:2. Prebiotic supplementation with GOS:FOS:lvPectin 9:1:2 had a high count in mesenteric lymphnodes of unchallenged mice but not in challenged mice. This preliminary screening did not result in any significant results but a cautiously optimistic alignment with other data warrants further investigation.

Figure 5 shows that prebiotic supplementation with GOS:FOS:lvPectin 9:1:2 had a high count in mesenteric lymphnodes of unchallenged mice (mean 12.5 with a PBS reference mean of 3.72) but not in challenged mice (mean 3.2 with a PBS reference mean of 5.48). High numbers of Tregs were also counted in the mice that received the synbiotic formula of *B Breve* M16-V and GOS:FOS:lvPectin 9:1:2 in the thoracic lympnodes (unchallenged mean 4.4; LPS challenged mean 0.862) with PBS reference mean of 0.5 in unchallenged mice and 0.3 in LPS challenged mice. Treg counts in spleen in the synbiotic group were also higher in unchallenged mice (mean 1.8) than in LPS challenged (mean 0.9), with a PBS reference mean of 0.6 in unchallenged mice and 0.5 in challenged mice. Statistical analysis did not reveal any significant results. Table 4 provides a full overview of the Treg data.

**Table 4:** Overview of FoxP3+ regulatory T cells in thoracic -, and mesenteric lymph nodes and spleen.

% CD4+ CD25+ FoxP3 cells								
	PBS–PBS		PBS–PRO		PBS-PRE		PBS-SYN	
	Mean	SEM	Mean	SEM	Mean	SEM	Mean	SEM
TLN	0.544	0.316	0.902	0.512	1.2	0.719	4.422	2.179
Treatment effect (fractional change PBS-PBS)	-		0.66		1.21		7.13	
MLN	3.72	2.24	9.167	0.406	12.5	5.48	6.56	3.081
Treatment effect (fractional change PBS-PBS)	-		1.46		2.36		0.76	
Spleen	0.6	0.173	1.133	0.233	0.24	0.051	1.84	0.787
Treatment effect (fractional change PBS-PBS)	-		0.89		−0.60		2.07	
	LPS–PBS		LPS–PRO		LPS-PRE		LPS-SYN	
	Mean	SEM	Mean	SEM	Mean	SEM	Mean	SEM
TLN	0.308	0.298	0.524	0.301	0.142	0.038	0.862	0.637
Challenge effect (fractional change PBS-PBS)	−0.43		-					
Treatment effect (% difference LPS-PBS)	-		70.1		−53.9		180	
MLN	5.48	3.335	-	-	3.2	1.859	3.82	2.536
Challenge effect (fractional change PBS-PBS)	0.47		-					
Treatment effect (% difference LPS-PBS)	-		n.a.		−41.6		−30.3	
Spleen	0.52	0.183	0.32	0.146	0.6	0.152	0.9	0.333
Challenge effect (fractional change PBS-PBS)	−0.13		-					
Treatment effect (% difference LPS-PBS)	-		−38.46		15.38		73.08	

### 3.6 Short Chain Fatty Acids

**Figure 6:**
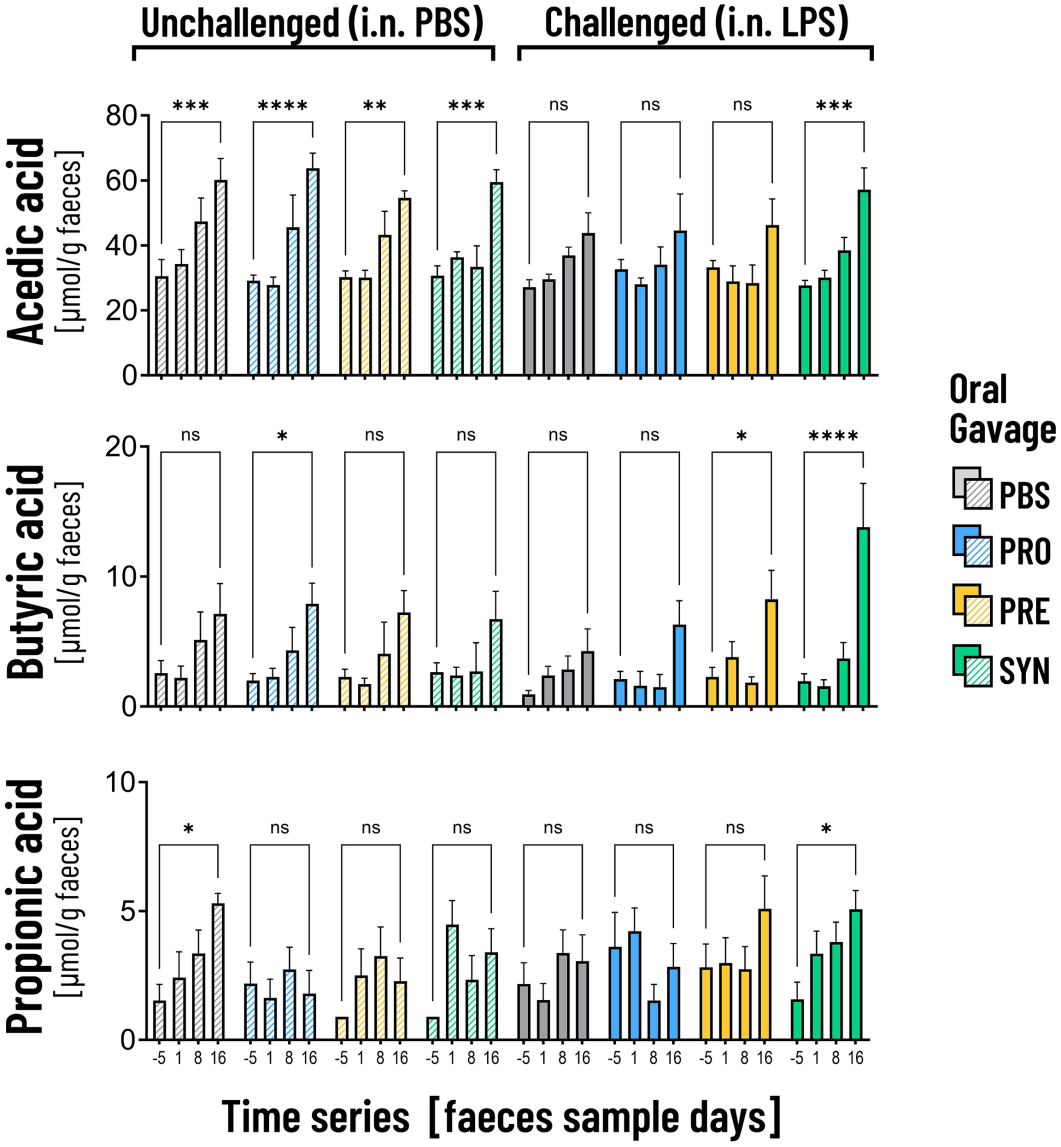
The graphs depict the analysis of acetic acid, butyric acid and propionic acid in fecal samples on 4 time points. Unchallenged mice (striped bars) showed a significant increase in the production of acetic acid over time (**p < 0.01; ***p < 0.001; ****p < 0.0001). In the LPS challenged mice the increase was only visible in mice that received the synbiotic mixture of B. breve M16-V and GOS:FOS:lvPectin (***p < 0.001). Mice that received this synbiotic mixture also had a significant increase in levels of butyric acid and propionic acid on day 16. The probiotic B. breve M16-V on itself only induced a significant increase in butyric acid in unchallenged mice. The prebiotic supplement GOS:FOS:lvPectin upregulated butyric acid in challenged mice but not acetic acid nor propionic acid.

**Figure 7:**
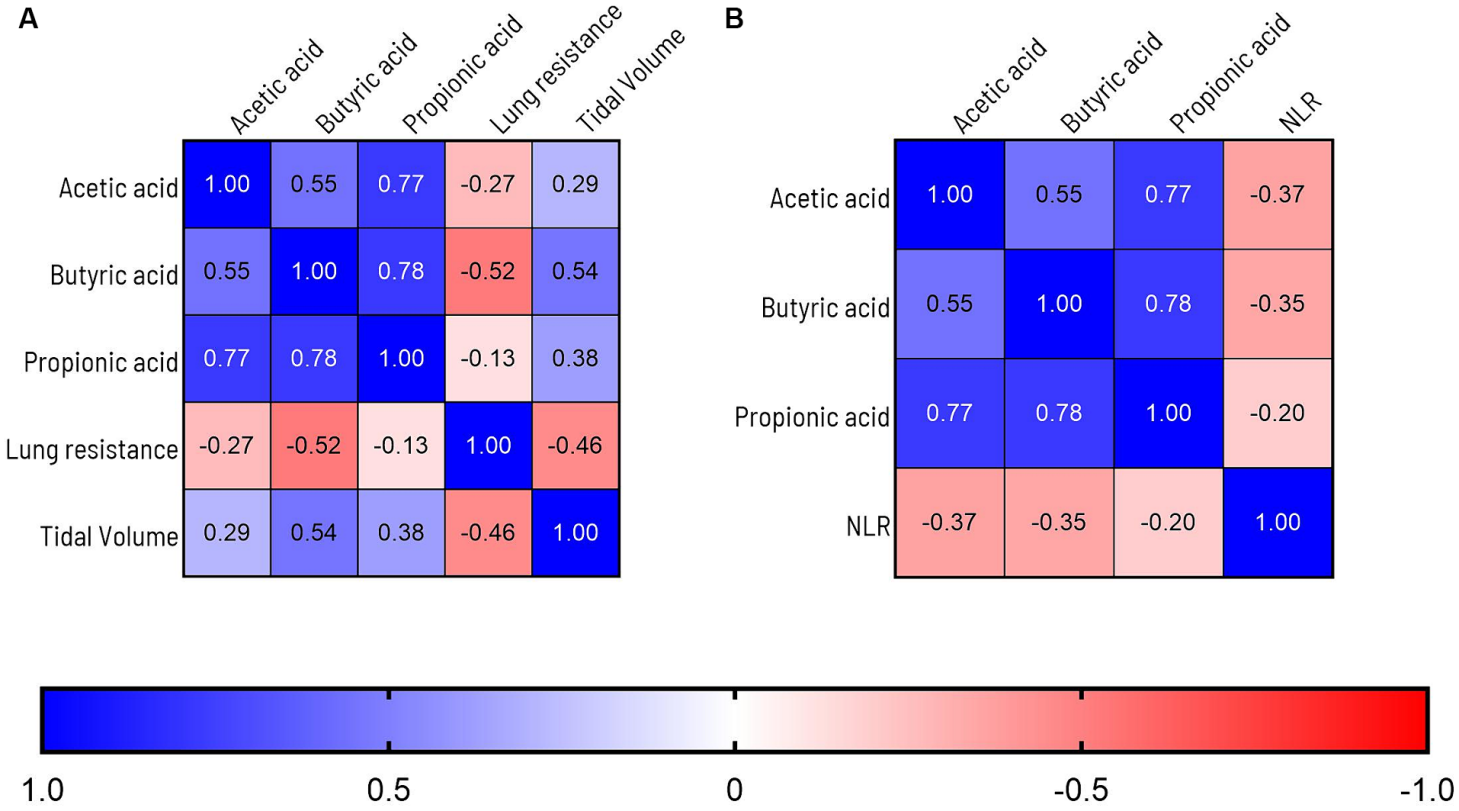
**(A)** Graph depicts Pearson correlations analysis of overlapping lung health and SCFA data points. A moderate correlation is observed between levels of butyric acid and improved lung function parameters. Levels of acetic acid were weakly correlated to improved lung resistance and tidal volume and propionic acid was moderately correlated to tidal volume and weakly to lung resistance. **(B)** Levels of acetic acid, butyric acid were moderately correlated to improvement of the BALF Neutrophil to Lymphocyte Ratio (NLR). To label the strength of the Pearson associations, the r values between, 0–0.3 (or −0.3) are considered as weak, 0.3–0.7 (or −0.7) as moderate, 0.7–1.0 (or −1) as a strong positive (or negative) correlations (85).

SCFA are suggested to support beneficial gut-lung crosstalk, therefore we analyzed concentrations of SCFA at 4 time points throughout the experiment (Figure 6). The first thing that stood out was that unchallenged mice showed a significant increase in the production of acetic acid between day −5 and day 16 (PBS-PBS *p* = 0.0003; PBS-Pro *p* < 0.0001; PBS-Pre, *p* = 0.0045; PBS-Syn, *p* = 0.0005) which in LPS challenged mice was only the case in the mice that received the synbiotic mixture of *B. breve* M16-V and GOS:FOS:lvPectin (*p* = 0.0003). Levels of acetic acid were 30.5% higher in LPS-synbiotic group compared to the LPS-placebo group. The fractional difference between day −5 and day 16 was 1.17 in the unchallenged placebo group and 0.73 in the challenged placebo group. Table 5 provides a full overview of the SCFA data. Similar patterns were observed for the amount of fecal butyric acid and propionic acid. All the analyzed enteric supplements induced higher levels of fecal butyric acid than the LPS-placebo group. On day 16, the levels of butyric acid in the synbiotic supplement group were 223.4% higher than day −5 (*p* < 0.0001), followed by 93.2% for the prebiotic group (*p* = 0.0229). The probiotic supplement group reached a 47.3% increase (n.s.). The levels of propionic acid were also significantly higher in the synbiotic group between day −5 and day 16 (65.9%, *p* = 0.0253) but not in any of the other LPS groups. To gain further insight into the meaning of observed fecal SCFA results, Pearson correlations were performed for mice in which both lung health and SCFA was measured simultaneously (*n* = 4). A moderate correlation was found between levels of butyric acid and improved lung function parameters (Figure 7A). Levels of acetic acid were weakly correlated to improved lung resistance and tidal volume and propionic acid was moderately correlated to tidal volume and weakly to lung resistance. Levels of acetic acid and butyric acid both showed a moderate negative correlation with pulmonary NLR (Figure 7B), which should be read as low levels of acetic acid and butyric acid corresponding to a higher pulmonary NLR.

**Table 5:** Overview of short chain fatty acid data measured in fecal samples.

Acetic acid	PBS–PBS		PBS–PRO		PBS-PRE		PBS-SYN	
	Mean	SEM	Mean	SEM	Mean	SEM	Mean	SEM
Day −5	30.51	5.14	29.10	1.77	30.27	1.89	30.66	3.03
1	34.27	4.45	27.80	2.45	30.14	2.20	36.34	1.68
8	47.40	7.19	45.57	9.94	43.24	7.23	33.39	6.50
16	60.13	6.62	63.76	4.63	54.66	2.16	59.49	3.84
Fractional difference within group (−5 versus 16)	1.17***	0.31	1.24****	0.23	0.87**	0.17	1.05***	0.22
% difference between group (PBS-PBS day 16)	-		6.0		−9.1		−1.1	
	LPS–PBS		LPS–PRO		LPS-PRE		LPS-SYN	
	Mean	SEM	Mean	SEM	Mean	SEM	Mean	SEM
Day −5	27.14	2.32	32.71	2.96	33.24	2.11	27.67	1.53
1	29.60	1.52	28.04	1.95	28.91	4.80	30.13	2.21
8	36.89	2.58	34.03	5.53	28.43	5.52	38.46	4.01
16	43.77	6.24	44.54	11.32	46.23	8.06	57.14	6.67
Fractional difference within group (−5 versus 16)	0.73 ^n.s.^	0.33	0.32 ^n.s.^	0.30	0.39 ^n.s.^	0.24	1.04***	0.18
% difference between group (LBS-PBS day 16)	-		1.8		5.6		30.5	

Butyric acid	PBS–PBS		PBS–PRO		PBS-PRE		PBS-SYN	
	Mean	SEM	Mean	SEM	Mean	SEM	Mean	SEM
Day −5	2.571	0.961	1.986	0.540	2.286	0.580	2.657	0.706
1	2.200	0.914	2.271	0.664	1.714	0.465	2.386	0.632
8	5.129	2.151	4.314	1.768	4.057	2.430	2.700	2.200
16	7.129	2.333	7.900	1.599	7.243	1.678	6.714	2.151
Fractional difference within group (−5 versus 16)	6.1 ^n.s.^	3.2	9.7*	4.5 ^n.s.^	7.3 ^n.s.^	4.2	2.8 ^n.s.^	1.9
% difference between group (PBS-PBS day 16)	-		10.8		1.6		−5.8	
	LPS–PBS		LPS–PRO		LPS-PRE		LPS-SYN	
	Mean	SEM	Mean	SEM	Mean	SEM	Mean	SEM
Day −5	0.943	0.291	2.100	0.606	2.29	0.72	1.94	0.57
1	2.371	0.716	1.600	1.100	3.80	1.20	1.54	0.51
8	2.843	1.045	1.486	0.986	1.84	0.44	3.69	1.24
16	4.267	1.705	6.286	1.860	8.24	2.23	13.80	3.36
Fractional difference within group (−5 versus 16)	5.9 ^n.s.^	3.7	7.2	3.7	4.3*	2.2	7.3****	1.4
% difference between group (LBS-PBS day 16)	-		47.3		93.2		223.4 #	

Propionic acid	PBS–PBS		PBS–PRO		PBS-PRE		PBS-SYN	
	Mean	SEM	Mean	SEM	Mean	SEM	Mean	SEM
Day −5	1.529	0.629	2.186	0.830	0.900	0.000	0.900	0.000
1	2.414	1.002	1.629	0.729	2.500	1.036	4.471	0.938
8	3.357	0.905	2.729	0.866	3.257	1.122	2.329	0.948
16	5.300	0.389	1.800	0.900	2.286	0.896	3.400	0.911
Fractional difference within group (−5 versus 16)	4.11*	0.93	0.76 ^n.s^	1.05	1.54 ^n.s^	1.00	2.78 ^n.s^	1.01
% difference between group (PBS-PBS day 16)	-		−66.04		−56.87		−35.85	
	LPS–PBS		LPS–PRO		LPS-PRE		LPS-SYN	
	Mean	SEM	Mean	SEM	Mean	SEM	Mean	SEM
Day −5	2.171	0.821	3.614	1.332	2.814	0.903	1.571	0.671
1	1.543	0.643	4.214	0.907	2.986	0.985	3.343	0.876
8	3.371	0.896	1.529	0.629	2.743	0.874	3.800	0.767
16	3.057	1.021	2.829	0.910	5.086	1.279	5.071	0.723
Fractional difference within group (−5 versus 16)	1.48 ^n.s^	1.06	0.47 ^n.s^	0.73	2.37 ^n.s^	1.22	3.98*	1.06
% difference between group (LBS-PBS day 16)	-		−7.46		66.37		65.88	

ns *p* > 0.05; *p ≤ 0.05; **p ≤ 0.01; ***p ≤ 0.001; ****p ≤ 0.0001.

## 4 Discussion

This study was focused on gaining more insight into gut-lung crosstalk in context of pulmonary neutrophilia. Based on existing literature describing immune activating and inhibitory effects along the gut-lung axis, both via innate TLR pathways, one could argue for health promoting as well as disease promoting potential of gut modulation in context pulmonary neutrophilia (35). *B. breve* however has been described as having a low pro-inflammatory profile, by not exerting TLR4 activation (74). Moreover the specific *B. breve* M16-V has a well-evaluated safety profile for food use (69). The current study provides further preclinical support for good tolerability of *B. breve* M16-V in context of hyperactivation of a key innate immune pathway, also when applied in a synbiotic combination with GOS:FOS:lvPectin 9:1:2. Vital signs and body weight measurements were not negatively influenced by any of the tested prophylactic formula containing *B Breve* M16-V and/or GOS:FOS:lvPectin 9:1:2.

Although gut microbiota can boost the defensive capacity of neutrophils in context of infection (34, 66, 67), none of the here evaluated gut modulation formula promoted an increase in the numbers of pulmonary neutrophils. In unchallenged mice the intra-gastric pro-, pre-, and synbiotic supplements did not induce influx of pulmonary inflammatory cells and in the LPS challenged mice all groups that received an enteric supplement had lower neutrophil counts compared to the LPS-placebo group. Of the analyzed enteric supplements the synbiotic supplement containing the combination of *B. breve* M16-V and GOS:FOS:lvPectin 9:1:2 had the most favorable effect on the LPS induced pulmonary neutrophila, defined by a significant decrease in numbers of neutrophils and a lower pulmonary NLR score. The differential cell analysis was only in part reflected in the cytokine profile. Both MIP and KC were significantly upregulated in challenged compared to unchallenged mice but in contrast to the cellular response the synbiotic treatment effect was observed for the MIP marker, reflecting macrophage activity and not for KC reflecting neutrophil chemotaxis.

It would be interesting to extent analysis to systemic measurements of cytokines, neutrophils and NLR. Blood NLR is a relatively easy to measure marker for systemic inflammation and incorporating this marker in future gut-lung studies can shed further light on the effect of diets on progression of severe inflammatory disorders (86). Furthermore, it will be interesting to perform differential analysis of lung and systemic immune cells including dendritic cells and effector T cells such as Th1, Th2, and Th17. More specifically it will be relevant to further examine CD4(+)Foxp3(+) regulatory T cells (Tregs), since these can be upregulated by gut microbiota and have been identified as having immune-suppressive effects beyond the gut (87). The preliminary exploration of the numbers of systemic Tregs was performed using thoracic lympnodes and spleen tissue of a small subset of the mice (supplementary data). The highest numbers of Tregs were counted in thoracic lympnodes and spleen of the unchallenged and challenged mice that received the synbiotic formula of *B. breve* M16-V and GOS:FOS:lvPectin 9:1:2. Prebiotic supplementation with GOS:FOS:lvPectin 9:1:2 had a high count in mesenteric lymphnodes of unchallenged mice but not in challenged mice. The means were however very spread out and significant results were not obtained, but a cautiously optimistic pattern does warrant further investigations. A follow-up study will be needed to further unravel if Tregs play a role in any of the observed beneficial effects of dietary supplements on lung health.

The beneficial effect of synbiotic mixture of *B. breve* M16-V and GOS:FOS:lvPectin 9:1:2 in context of pulmonary neutrophilia was also reflected in the significantly improved functional parameter of lung resistance. It was interesting to see that in contrast to the individual data points, the linear regression analysis of the slope patterns also provided indications for a treatment effect of probiotics, *p* < 0.0001 and prebiotics *p* = 0.0042. Further analysis is however needed to see if this trend will result in a more profound effects of probiotics and prebiotics in a more stressful setting than the here used highest methacholine challenge.

In addition to lung inflammation and lung function, lung morphology was analyzed also. Of the LPS challenged mice the prebiotic and synbiotic group presented less, however not a significant decrease in the disruption of alveolar walls compared to the LPS-placebo group. A previous study did show that *B. breve* has the ability to inhibit alveolar damage but in that specific study the amount of neutrophils was not affected by *B. breve*, in contrast to the prebiotic GOS/FOS fiber mixtures that did attenuate both neutrophil influx and alveolar damage (76). Comparing these LPS studies is hampered by the differences in study design. In the current study 7 i.n doses of LPS were spread over a time frame of 15 days with the section 1 day after the final challenge whereas the previous study used 8 doses spread over 24 days with the section 4 days after the final LPS challenge (76). Compared to the former study, the shorter timelines of the current study were better suited for studying effects on overwhelming innate immune activation (e.g., pulmonary neutrophil infiltration). The more stretched time-period of the previous study however provided a better window for analyzing the dietary effect on changes in the lung architecture, with a consequence of having a limited window of studying the effect on innate immune activation.

Another novel insight from the current LPS model compared to the previous LPS model, was that i.n. delivered LPS caused an attenuation in the production of fecal SCFAs over time, compared to the control mice that received PBS (37.6% less increase of acetic acid and 64% less increase of propionic acid). Moreover enhanced levels of butyric acid, acetic acid and propionic acid, following supplementation with synbiotic mixture of *B. breve* M16-V and GOS:FOS:lvPectin, associated with better lung health. This finding suggests bidirectional gut lung crosstalk involving microbial derived metabolites which confirms previous proposed links between microbes, metabolites, and gut-lung health (30, 88, 89). In other rodent models it has also been shown that common triggers for pulmonary inflammation, such as cigarette smoke and environmental particulate matter can cause intestinal inflammatory responses, alter the gut microbiota and trigger decreased SCFA production (90, 91). Moreover in an elastase induced model for emphysematous inflammation it was previously shown that increased cecal SCFA coexisted with improved lung health that was induced by a diet, more specifically a whey peptide-based enteral supplement (82).

The exact timelines of SCFA production and mechanisms of the lung driven effect on gut microbiota derived metabolites, are not yet elucidated. In the current study mice were relatively young, 6–8 weeks and we speculate that alongside their normal weight gain, the gut microbiota was still developing explaining the SCFA gain. Building on insights from other investigators one could speculate that LPS spilling over from the lungs could have a direct negative effect on the gut microbiota. Supportive of this idea is the finding that LPS that is cleared from systemic circulation causes intestinal inflammation driven by the different localization of LPS in intestinal epithelial cells and in more differentiated enterocytes (92). In contrast to systemic derived LPS, the presence of luminal LPS from commensal gut bacteria, is usually well tolerated by the mucosal immune system which can in part be explained by TLR4 subcellular distribution and ligand-specific dynamic regulation (40). An indirect mechanism of action could also be speculated upon, since other investigators showed that acute instillation of LPS in mouse lungs changes bacterial microbiota in the lungs, and causes an increase in number of bacteria in blood and gut (93).

More support for an indirect lung-gut crosstalk can be found in a study in mice showing that airways infected with an influenza virus can cause outgrowth of *E. coli* combined with abnormal inflammatory responses involving T-helper 17 cells and intestinal damage. These effects were explained via migrating lung derived CC-chemokine receptor 9 positive (CCR9^+^)CD4^+^ T cells (94). Intestinal injury was also observed secondary to pneumonia (95). The specific effect of neutrophilic lung diseases, such as COPD, on changes in gut microbiota has not been thoroughly studied (27). Investigators did report that smoking, which is a key risk factor for pulmonary neutrophilia, affects intestinal microbiota and smokers specifically express decreased abundance of *Bifidobacterium* spp. (27, 96, 97). Moreover, *in vitro* and *in vivo* studies showed that cigarette smoke has a negative effect on the production of SCFAs by *Bifidobacterium* (90, 98). In light of this data, the current study adds encouraging insight by showing that the synbiotic supplement containing *B. breve* M16-V and GOS:FOS:lvPectin 9:1:2 was capable of preventing the decreased SCFA production ánd improving lung health. Taken together the findings from the current study emphasize the potential benefit of bidirectional gut lung cross-talk in context of pulmonary neutrophilia.

## 5 Limitations and recommendations

Apart from fecal SCFA analysis the current study is limited in gut ecosystem analysis. In a follow up study it will be interesting to analyze intestinal inflammation and the composition of the intestinal microbiome before and after LPS treatment and nutritional supplementation. This would help to shed further light on the relation between lung inflammation and fecal SCFA production. Moreover, it is recommended to assess systemic-, and lung SCFA concentrations, in addition to fecal SCFA levels. In addition a recent study pointed toward the value of analyzing pulmonary TLR expression levels in explaining a beneficial effect of probiotics in context of lung disease (99). The specific study showed that mRNA expression levels of TLR2, TLR4 and TLR9 were all upregulated in lung tissue in response to cigarette smoke which was reduced significantly by oral feeding with *Lactobacillus rhamnosus*.

Although the LPS trigger in the current study was strategically chosen as a robust and reproducible stressor, activating an innate immune pathway that is broadly relevant for neutrophilic lung disorders including COPD, ARDS and persistent neutrophilic asthma (44–53), it does have limitations. It should be acknowledged that such persistent airway diseases can involve more complex mixtures of stressors (e.g., cigarette smoke, air pollutants and bacterial or viral infections) driving simultaneous activation of multiple inflammatory cascades. It must also be realized that although valuable insights can be obtained from rodent models (e.g., lung function and gut-lung interactions), there are limitations in cross-species translation. Significant differences exist between mouse and human lung architectures, their digestive and immune systems (100–102). Further research is thus warranted to investigate whether the here presented lung health improving effects of the synbiotic mixture of *B. breve* M16-V and GOS:FOS:lvPectin, can be reproduced in humans and in context of more complex mixtures of stressors, such as cigarette smoke, air pollutants and hyperinflammatory infectious disorders.

It is also important to realize that the here presented study describes the effect of prophylactic supplements which translates to prevention of hyperinflammation rather than management of existing chronic inflammatory disorders. To overcome this limitation, it is recommended to include the analysis of safety and effectiveness of microbiota manipulation after onset of dysregulated lung inflammation. For further analysis of the effect and underlying mechanisms of gut modulation on emphysematous disease, it is also recommended to perform longer studies, allowing for more gradual alveolar wall breakdown that better represent the long timelines of disease progression in humans. Furthermore, it is relevant to elaborate investigations on the effect of lung disorder progression on the composition of gut microbiota and metabolites. The bidirectional effect of lung diseases on gut microbiota and metabolites composition are most ideally analyzed using human material since this will provide more ethical and relevant guidance of insights on safety and effectiveness of gut microbiota modulating therapies and its time-frames of delivery.

A final limitation of the current study that was identified is the absence of a reference drug. It is thus advised to compare the effects of gut microbiota manipulation on lung health and disease with reference medications and in combination with standard of care treatments.

## 6 Concluding remarks

Despite shortcomings, the here presented work provides relevant insight on bi-directional gut-lung crosstalk in context of pulmonary neutrophilia (Graphical abstract). Repeated pulmonary challenge with LPS induced pulmonary neutrophilia and negatively impacted fecal SCFA concentrations. The study provided proof of concept for tolerability and effectiveness of the synbiotic combination of *B. breve* M16-V and scGOS/lvFOS/lvPectin to prevent the LPS-induced decline in fecal SCFA levels and to dampen the development of LPS induced pulmonary neutrophilia, to improve pulmonary NLR and to dampen lung function decline. Targeting neutrophils has recognized potential in the management of severe acute and poorly controlled chronic inflammatory airway disorders (103, 104). The observed beneficial lung effects associated with enhanced SCFA production. Taken together, this experiment provided supportive data for the value of SCFA producing synbiotic nutritional concepts, more specifically the mixture of *B. breve* M16-V and scGOS/lvFOS/lvPectin, to prevent adverse functional consequences in context of neutrophilic lung disorders.

## Data availability statement

The original contributions presented in the study are included in the article/supplementary material, further inquiries can be directed to the corresponding authors.

## Ethics statement

The animal study was approved by Utrecht Universities Committee on Animal Research. The study was conducted in accordance with the local legislation and institutional requirements.

## Author contributions

GB: Conceptualization, Data curation, Formal analysis, Investigation, Methodology, Project administration, Validation, Visualization, Writing – original draft, Writing – review & editing. MD: Data curation, Investigation, Writing – review & editing. EM: Data curation, Investigation, Writing – review & editing. IA: Data curation, Formal analysis, Investigation, Methodology, Writing – review & editing. JB: Writing – review & editing. AK: Conceptualization, Funding acquisition, Supervision, Writing – review & editing. GF: Conceptualization, Funding acquisition, Methodology, Supervision, Writing – review & editing. JG: Conceptualization, Funding acquisition, Supervision, Writing – review & editing.

## Glossary

ALI: Acute lung injury
ANOVA: Analysis of variance
APC: Allophycocyanine
ARDS: Acute respiratory distress syndrome
BAL: Broncho alveolar lavage
BALF: Broncho alveolar lavage fluid
*B. breve*: *Bifidobacterium breve*
BSA: Bovine serum albumin
BW: Body weight
CAMPs: Commensal associated molecular patterns
CBA: Cytometric bead array
COPD: Chronic obstructive pulmonary disease
FACS: Fluorescence activated cell sorting
FITC: Fluorescein isothiocyanate
GC: Gas chromatograph
GM-CSF: Granulocyte macrophage-colony stimulating factor
GOS:FOS:lvPectin: Short-chain galactooligosaccharides, long-chain fructo-oligosaccharides, and low-viscosity pectin
H&E: Hematoxylin/eosin
IgG: Immunoglobulin G
IL23 P19/P40: Mouse interleukin-23 (p19/p40)
i.n: Intra nasally
SCFA: Short chain fatty acids
scGOS/lcFOS: Short-chain galacto-oligosaccharides and long-chain fructo-oligosaccharides
KC: Keratinocyte-derived chemokine
Lm: Mean linear intercept
LPS: Lipopolysaccharides
MAMPs: Microbial associated molecular patterns
MFI: Mean fluorescence intensity
MIP1a: Macrophage inflammatory protein-1α
MLN: Mesenteric lymph nodes
NLR: Neutrophil to lymphocyte ratio
n.s.: Not significant
PAMPs: Pathogen associated molecular patterns
PBS: Phosphate-buffered saline
PE: Phycoerythrin
R_L_: Lung resistance
SEM: Standard error of mean
Th: T helper cell
TLN: Thoracic lymp nodes
TLR: Toll like receptor
Tregs: CD4(+)Foxp3(+) regulatory T cells
V_t_: Tidal volume
